# Identification and characterization of genes associated with tapping panel dryness from *Hevea brasiliensis *latex using suppression subtractive hybridization

**DOI:** 10.1186/1471-2229-10-140

**Published:** 2010-07-09

**Authors:** Dejun Li, Zhi Deng, Chunliu Chen, Zhihui Xia, Min Wu, Peng He, Shoucai Chen

**Affiliations:** 1 Key Laboratory of Rubber Biology, Ministry of Agriculture, Rubber Research Institute, Chinese Academy of Tropical Agricultural Sciences, Danzhou, Hainan 571737, China; 2 Hainan Provincial Key Laboratory of Tropical Crops Cultivation and Physiology, Rubber Research Institute, Chinese Academy of Tropical Agricultural Sciences, Danzhou, Hainan, 571737, China; 3 Institute of Biological Science and Technology, College of Agriculture, Hainan University, Haikou, 570228, China

## Abstract

**Background:**

Tapping panel dryness (TPD) is one of the most serious threats to natural rubber production. Although a great deal of effort has been made to study TPD in rubber tree, the molecular mechanisms underlying TPD remain poorly understood. Identification and systematical analyses of the genes associated with TPD are the prerequisites for elucidating the molecular mechanisms involved in TPD. The present study is undertaken to generate information about the genes related to TPD in rubber tree.

**Results:**

To identify the genes related to TPD in rubber tree, forward and reverse cDNA libraries from the latex of healthy and TPD trees were constructed using suppression subtractive hybridization (SSH) method. Among the 1106 clones obtained from the two cDNA libraries, 822 clones showed differential expression in two libraries by reverse Northern blot analyses. Sequence analyses indicated that the 822 clones represented 237 unique genes; and most of them have not been reported to be associated with TPD in rubber tree. The expression patterns of 20 differentially expressed genes were further investigated to validate the SSH data by reverse transcription PCR (RT-PCR) and real-time PCR analysis. According to the Gene Ontology convention, 237 unique genes were classified into 10 functional groups, such as stress/defense response, protein metabolism, transcription and post-transcription, rubber biosynthesis, etc. Among the genes with known function, the genes preferentially expressed were associated with stress/defense response in the reverse library, whereas metabolism and energy in the forward one.

**Conclusions:**

The genes associated with TPD were identified by SSH method in this research. Systematic analyses of the genes related to TPD suggest that the production and scavenging of reactive oxygen species (ROS), ubiquitin proteasome pathway, programmed cell death and rubber biosynthesis might play important roles in TPD. Therefore, our results not only enrich information about the genes related to TPD, but also provide new insights into understanding the TPD process in rubber tree.

## Background

Rubber tree (*Hevea brasiliensis *Muell. Arg.) is a perennial tropical tree for the production of natural rubber (NR). In the world, at least 2000 plant species are recognized for producing latex, but the rubber tree is the only economically viable source of NR. Rubber molecules are produced, aggregated and packaged in the latex vessels (laticifers) of rubber tree. The latex, a cytoplasmic component of the laticifers, expels from the laticifers upon tapping. Over the past decades, the rubber yield has been significantly increased, due to the cultivation of high-yielded clones and the utilization of ethephon (an ethylene generator). However, latex production still faces serious economic losses caused by TPD. At present, there are no effective measures to prevent or treat TPD in rubber tree. It was estimated that the losses due to TPD accounted to 12-14% of the annual rubber production [1].

The first symptom of TPD is the appearance of partial dry zones (no latex flow) along the tapping panel. In the advanced stage, the tapping panel may even become completely dry and other symptoms such as browning, thickening, or even flaking of bark can occur [2]. A great deal of work has been done to reveal the nature and molecular mechanisms of TPD. It was initially thought that TPD might be caused by pathogens [3-5], but no further evidence has been found to support this claim [6,7]. Physiological studies suggested that the TPD syndrome was a complex physiological disorder resulted from over tapping and overexploitation (excessive tapping as well as overstimulation with ethylene) [8-12]. During the process of TPD, the lutoids burst and consecutive in situ latex coagulation caused by membrane destabilization, which has been proposed to be associated with the occurrence of an uncompensated oxidative stress within the latex cells [8]. In TPD tree, the contents of protein, nucleic acid, thiols and ascorbic acid decreased [10], whereas the activities of RNase and proteinase increased in general [13-15]. In addition, the levels of variable peroxidase and superoxide dismutase (SOD) also decreased [16]. Through proteomics, some researchers have identified proteins related to TPD by comparing the expression patterns between healthy and TPD trees [2,17-19], but their functional relations with TPD still remain unknown.

Besides the reports mentioned above, the identification and characterization of genes associated with TPD also made some progresses in rubber tree. Our group reported a key transcription factor, *HbMyb1*. Compared with healthy tree, the expression of *HbMyb1 *was significantly decreased in barks and latex of TPD tree [20]. Functional analyses further indicated that *HbMyb1 *negatively regulated programmed cell death (PCD) in transgenic tobacco plants (unpublished data). Venkatachalam et al. identified 134 genes associated with TPD in rubber tree by SSH method. Moreover, they analyzed the expression patterns of partial genes and discussed the relationship between differentially expressed genes and TPD [21]. Two years later, they identified a gene, *HbTOM20*, associated with TPD by mRNA differential display. *HbTOM20 *might play an important role in the alteration of mitochondrial metabolism, which finally resulted in impaired latex biosynthesis [22].

Although significant progress has been made on TPD, the molecular mechanism underlying TPD still remains largely unknown. Identifying the genes related to TPD and analyzing their expression patterns are the prerequisites for elucidating the molecular mechanism involved in TPD. Venkatachalam et al. has identified the expression profiles of the genes related to TPD from *Hevea *latex [21]. It is necessary to identify the genes related to TPD with different rubber clones since TPD is genetically determined [10]. Overstimulation with ethylene may result in TPD [8-12], so it is reasonable for identifying the genes associated with TPD to select the rubber trees with ethylene stimulation as materials. At present, the data on genes related to TPD is scarce; the molecular mechanism underlying TPD could be precisely elucidated only if enough the genes related to TPD were identified. With these considerations, we designed the experiments to identify the genes associated with TPD, and then further analyzed the functional categories and expression patterns of the genes associated with TPD. Combined with the results from Venkatachalam et al., the potential pathways involved in TPD are discussed.

## Results

**Figure 1:**
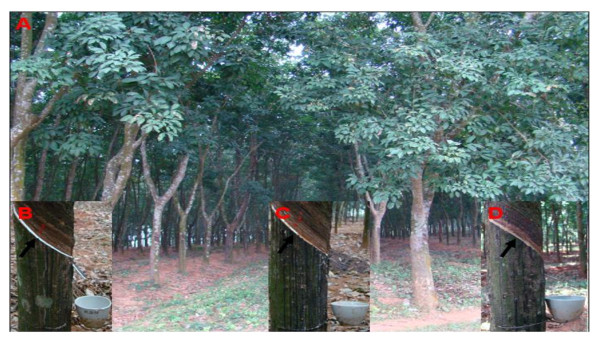
**The growth states of an elite rubber clone (RY8-79)**. (A) The plantation of an elite rubber clone (RY8-79) at the experimental farm of Chinese Academy of Tropical Agricultural Sciences. (B) A healthy tree with normal latex flow (indicated by arrow). (C) A rubber tree partially affected by TPD in which latex flow is observed in patches (indicated by arrow). (D) A rubber tree completely affected by TPD in which no latex flow is observed (indicated by arrow).

To identify the genes related to TPD, subtracted cDNA libraries were constructed. In this experiment, the elite clone (RY8-79) with ethylene stimulation was selected as experimental material; the latex has been harvested from these trees for the past 11 years (Figure 1A). The healthy and TPD rubber trees were selected according to the phenotypes. After 11 years of exploitation, more than 80% of the rubber trees had normal latex flow after tapping (Figure 1B) while the remaining trees had a partial or complete stoppage of latex flow (Figure 1C and 1D). The trees with normal latex flow were considered as "healthy" trees, whereas the trees with a partial or complete stoppage of latex flow were referred to as "TPD" trees. As for TPD trees, the trees showing a partial and complete stoppage of latex flow were defined as the initial and advanced stages of TPD, respectively. In this research, the latex was collected from the TPD tree at initial stage (Figure 1C). To capture a wide spectrum of differentially expressed genes, latex samples were collected and pooled from 5 different trees for mRNA isolation and cDNA library construction.

Two subtracted cDNA libraries were successfully generated. One, "forward" library from healthy trees, was subtracted by the cDNA pool of TPD trees; the other, "reverse" one, was obtained from TPD trees subtracted by the cDNA pool of healthy trees. In total, 1,106 positive colonies containing cDNA inserts were identified by PCR detection from the forward and reverse libraries.

### Evaluation of subtractive efficiency

**Figure 2:**
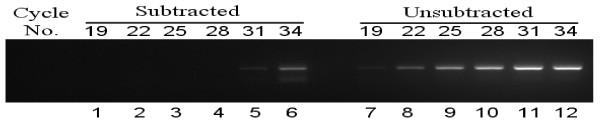
**The detection of subtraction efficiency by PCR**. PCR was performed on the subtracted (lanes 1-6) and unsubtracted (lanes 7-12) samples with 18s rRNA primers. The numbers of PCR cycles are indicated above the panel.

It is well-known that the subtraction efficiency is vital for the successful construction of subtracted cDNA libraries. In the research, the subtraction efficiency was evaluated by amplifying rubber 18s rRNA gene (a house keeping gene). If the subtraction process is efficient, the transcripts of 18s rRNA gene should be reduced. As shown in Figure 2, the 18s rRNA PCR products appeared to be detectable after 19- and 31-cycle amplifications with unsubtracted and subtracted cDNA as PCR templates, respectively. Compared with the unsubtracted samples, the abundance of rubber 18s rRNA was sharply decreased in subtracted ones, which indicated that the samples were effectively subtracted. Therefore, it was expected that the genes differently expressed between healthy and TPD trees were enriched in two cDNA libraries.

### Reverse Northern analysis of the cDNA clones identified by SSH

**Figure 3:**
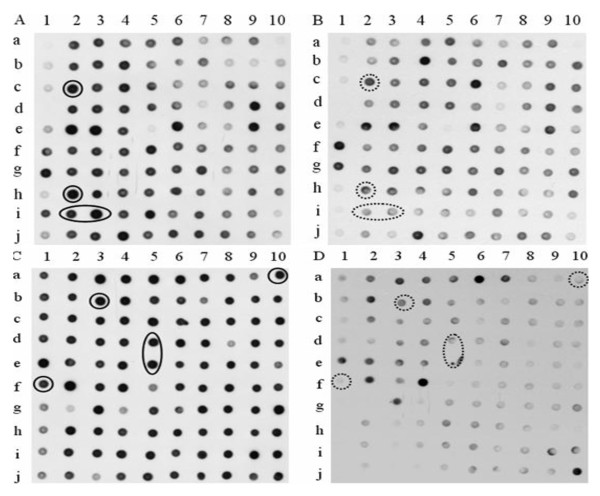
**The differential screening of cDNA clones from rubber SSH libraries**. Duplicate dot-blots were prepared and the membranes were hybridized with labeled probes. The spots displaying different hybridization signals between two membranes were classified as either up- (circled by continuous line) or downregulated (circled by dotted line). (A) The clones from forward SSH library hybridized with unsubtracted tester probes. (B) The clones from forward SSH library hybridized with unsubtracted driver probes. (C) The clones from reverse SSH library hybridized with unsubtracted tester probes. (D) The clones from reverse SSH library hybridized with unsubtracted driver probes.

Although SSH is a powerful method for identifying differentially expressed genes, the subtractive samples may still contain some cDNAs that correspond to mRNA common to both tester and driver samples. In order to exclude these clones before sequencing, the cDNA positive clones from two cDNA libraries were further hybridized with different probes, first with the unsubtracted probes and then with the subtracted ones. For the forward library, the clones, showing strong hybridization signals with the unsubtracted cDNA probes from healthy trees (Figure 3A) and weak hybridization signals with the unsubtracted cDNA probes from TPD trees (Figure 3B), were considered as specific cDNAs upregulated in healthy tree. For the reverse library, the clones, indicating strong hybridization signals with the unsubtracted cDNA probes from TPD trees (Figure 3C) and weak hybridization signals with the unsubtracted cDNA probes from healthy trees (Figure 3D), were referred to as specific cDNAs upregulated in TPD tree. The clones, indicating the similar hybridization signals with the unsubtracted cDNA probes from TPD and healthy trees (Figure 3), were considered as common cDNAs that did not differentially expressed between healthy and TPD trees. Similarly, the secondary screening was also performed with subtracted probes (data not shown). This screening step made it possible to effectively eliminate the clones that show common expression in subtracted samples.

After the screening steps mentioned above, a total of 822 clones differentially expressed in two cDNA libraries were selected to sequence. The vector and adaptor sequences were firstly removed from the sequencing results, and then the poor-quality sequences were deleted. Sequence analyses indicated that the 822 clones represent 237 unique genes. The EST redundancy rate in this study was about 71.2%. All the sequences were deposited in the NCBI databases [GenBank: GO349116-GO349154, GO349156-GO349349 and GO788493-GO788496]. Among 237 unique genes, 162 and 75 were from the reverse and forward SSH libraries, respectively (Additional file 1 and 2).

### Annotation and functional classification of ESTs

237 unique ESTs were analyzed with the Blast program from the NCBI database and the information was displayed in Additional file 1 and 2. The results of the Blastn searches indicated that about 94.9% of unique ESTs matched with the known sequences in NCBI ESTs database; the remaining ESTs showed 'no hits found'. Among 237 unique ESTs, only 49 (about 20.7%) were aligned to known EST sequences from rubber tree, that is to say, 188 were firstly identified in rubber tree. In addition, the 237 unique ESTs were also analyzed with Blastx program in NCBI non-redundant protein database. As shown in Additional file 1 and 2, about 90.7% of ESTs had high similarities with the known proteins. Among these known proteins, only 8.9% were from rubber tree.

**Figure 4:**
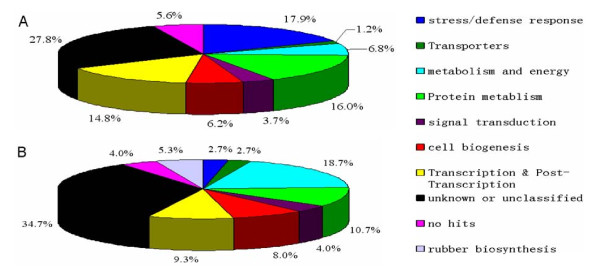
**The functional distributions of 237 ESTs from the reverse (A) and forward (B) libraries**. The classification was performed according to GO convention. The percentage of genes in each group was listed, and the legend was showed in right.

Based on the similarities to the known proteins, the functional classification was performed according to the Gene Ontology (GO) convention [23]. The 237 unique genes were classified into 10 functional categories such as stress/defense response, transporter, metabolism and energy, signal transduction, etc (Figure 4). The functional diversity of the genes related to TPD suggested that TPD might be a complex biological process. As shown in Figure 4, the reverse library contained all functional classifications from forward one except for rubber biosynthesis (RB). In the two libraries, the major classification group was the genes with unknown or unclassified roles; the percentages were about 27.8% and 34.7% in the reverse and forward libraries, respectively. Among the genes with known function, the stress/defense response genes made up the biggest group, followed by genes associated with protein metabolism, transcription and post-transcription in the reverse library (Figure 4A). In the forward library, the largest classification group was metabolism and energy, followed by protein metabolism and transcription and post-transcription (Figure 4B). Compared with healthy tree, the genes associated with stress/defense response were largely upregulated; whereas the genes related to metabolism and energy were largely downregulated in TPD tree (Figure 4), suggesting that these genes might play very important roles in TPD process.

### Validation of SSH data

**Figure 5:**
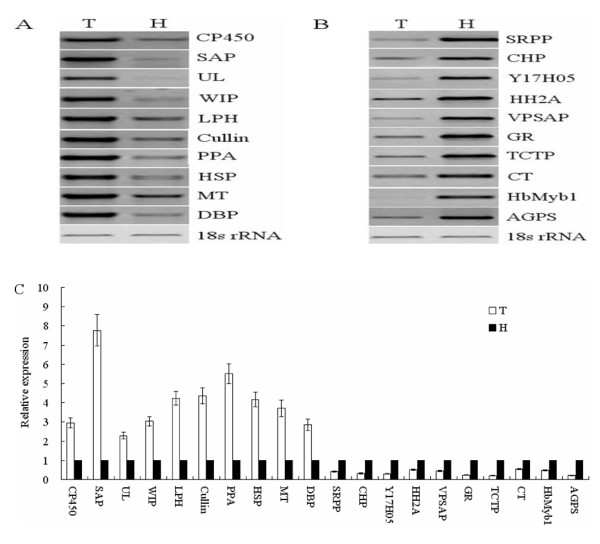
**The expression analyses of differently expressed genes by RT-PCR and real-time PCR**. Total RNA extracted from the latex was used for RT-PCR and real-time PCR analyses with the 18s rRNA as the internal reference. H and T represent healthy and TPD trees, respectively. The gene-specific primers were provided in Table 1. (A) The RT-PCR analyses of the genes upregulated in TPD tree. (B) The RT-PCR analyses of the genes upregulated in healthy tree. (C) The relative expression levels of the genes related to TPD in healthy and TPD trees. The data were collected from the real-time PCR analyses and shown as averages ± SE. The expression level of each gene in healthy tree was defined as 1.0.

To validate the genes differentially expressed between the two libraries, 10 genes from the forward library and 10 genes from the reverse one were selected to perform the expression analyses with RT-PCR and real-time PCR. These genes were associated with signal transduction, metabolism and energy, RB, unknown function, stress/defense response, transcription and post-transcription, protein metabolism, transporter and cell biogenesis. The specific primers of these 20 genes are listed in Table 1. As shown in Figure 5A and 5B, all genes from the forward library were downregulated in TPD tree, whereas all genes from the reverse one were upregulated in TPD tree. In addition, the expression levels of these genes were also quantified by real-time PCR analysis; all the data from real-time PCR analysis coincided with the results obtained from RT-PCR analysis. Among these genes, the SAP transcripts in TPD tree were approximately 7.8-fold than that in healthy tree; the translationally controlled tumor protein (TCTP) transcripts in TPD tree were about 0.2-fold that in healthy tree (Figure 5C). The expression profiles of the 20 genes sufficiently validated the reliability and accuracy of SSH data in this research. Moreover, the results presented here further demonstrated that the subtracted libraries might contain the genes differently expressed between TPD and healthy trees.

**Table 1:** The primers of genes detected by RT-PCR and real-time PCR analyses.

**Putative Genes**^**a **^**(Accession No.)**	RT-PCR primers (5'-3')	Real-Time PCR primers (5'-3')
WIP (GO349116)	F:GACATCGTATCATCAGGAAAAG	F: CGTATCATCAGGAAAAGTTGG
	R: CTCTCACCTGGGTAATTATC	R: GCTAGAATCGCAGCCTTCAG
SAP (GO349117)	F: GAATACGAACCGTGAAAGCG	F: GTGGCCTATCGATCCTTTAG
	R: CTAGGTTGAATTACCATCGC	R: ACGTCGCTATGAACGCTTGG
HSP (GO349120)	F: TTGTCCCTTAGAGCTTGTG	F: GTTGTCCCTTAGAGCTTGTG
	R: CTCCACCAAGAGTGGTGATG	R: GACCGTGTTGTTGATTCTCC
MT (GO349141)	F: ACCAAGGCAGAACTGAAGG	F: CACCAAGGCAGAACTGAAGG
	R: CCACTTTCTGATTCCTCCTC	R: CTGGATGTAGGATTCATCGG
CP450 (GO349147)	F: GACAAGGGGAATCCGACTG	F: CAATGTGATTTCTGCCCAGTG
	R: CCACACGAGATTTCTGTTCTC	R: TGACGAGGCATTTGGCTACC
Cullin (GO349159)	F: GTCCAATTGGTAATGCTTAAAC	F: CCTGCTCAAGCTATCCCTTC
	R:GGAAAGAGCCCATGAGTAAAG	R: GTCACGATTTCTGGCCAACC
UL (GO349162)	F: AGGATTAGACTTGACATAGCG	F: GATGGCCGATGGTTGAAAATG
	R: CTCCAATTTATCAGTGCCGC	R: CTGGATCTGAGTTTGCTGTTG
PPA (GO349189)	F: GTTGAACCGGAGGAATTTCC	F: GAATCGGATTCGTGGAAGTG
	R: TCATGGCCAGAAATGTCTCC	R: CTGTCCCTCAATCTCAAAAGG
LPH (GO349193)	F: TGTGCCACCAAGGTGCAAAC	F: GCAAGGGATCCAGGTTCATC
	R: GTCGTGGCAAACATACGTTG	R: CACCTTATGTGCGACATCGAC
DBP (GO349205)	F: TGAACTGGAACAGAGCAAGC	F:GATGAACTGGAACAGAGCAAG
	R: TTGACCCCGAACAATCTCAG	R: CTGTTCACGTTCTTTCAGAAC
TCTP (GO349268)	F: GCCTCCATCAGCGTTTTCAG	F: GTGTCAACAACTTGATGAACC
	R: CCCTCAATGATATCGACACC	R: ATGAGGGTGTTGATGACCAG
SRPP (GO349270)	F: CTGAAGAGGTGGAGGAAGAG	F: CCTTTATGCCAAGGACATATC
	R: CAGAGCTTTTGCGCCTTCCT	R: GTCTACAAACTTGACAGCCTC
AGPS (GO349278)	F: TTCTCACTCTCTCACGATGG	F: CCATACAATCTTACACTCACC
	R: CGAGGTTCATTCCACACATC	R: CTATGTCCAGCTCGTCCTTC
HH2A (GO349279)	F: AAGAATTCCGCGGCCTCCTG	F: GAAGCCAGTTTCTAGGTCTG
	R:ACTCGAGAACGGCAGCCAAG	R: GGCAGCCAAGTAAACTGGAG
CT (GO349296)	F: ATCCTTCTCCTGATCTCCTC	F: TTCTCCTGATCTCCTCCAAG
	R: GGGAGCGAGAGGAAGCTAG	R: CAAACAAGAGCGTTACCTCG
GR (GO349295)	F: TGAGGCATTGAGTCTGGAAG	F: TGAGGCATTGAGTCTGGAAG
	R: TGCTCTATTGCCTGCTCTTC	R: GAAAACTAGGTCCACAGTGG
VPSAP (GO349303)	F: ATTGTATGAGGACTCGAGATC	F: GAGGACTCGAGATCAAAGTG
	R:AGAACTTTGCTGAGCTCTATG	R:GAAGACAAAGATGAAAGAGTGG
HbMyb1 (GO349308)	F: AGGATGAACCTGATCGATGG	F: CCAGACACCAAGTCTCCTTC
	R: CTTCTCTCATCCTTCCCTCC	R: ATCCTTTGGCCATGCCAACC
CHP (GO349321)	F:CTCAACAACTCCAGTTGGTG	F: TCTTCAAAGCTCAACAACTCC
	R: ACCACTTCGACATATCCTCC	R: TTGCAGGCTCTCAATTGCTC
Y17H05 (GO349330)	F:GTGATGCATGATGTGAGGAG	F: TGGTGATGCATGATGTGAGG
	R:GAAATCCTAGGGCATCATTG	R: GTAAGACCATTGGACAAACGG
18s rRNA gene (AB268099)	F: GGTCGCAAGGCTGAAACT	F: GCTCGAAGACGATCAGATACC
	R: ACGGGCGGTGTGTACAAA	R: TTCAGCCTTGCGACCATAC

^a ^WIP, SAP, HSP, MT, CP450, UL, PPA, LPH, DBP, TCTP, SRPP, AGPS, HH2A, CT, GR, VPSAP, HbMyb1, CHP and Y17H05 represent Wound-induced protein, Senescence-associated protein, Heat shock protein, Metacaspase type II, Cytochrome P450-like TBP protein, Ubiquitin ligase, Posphotyrosyl phosphatase activator, Latex profilin Hev b8, DNA-binding protein RAV1, Translationally controlled tumor protein, Small rubber particle protein, Alpha-1,4-glucan-protein synthase, Histone H2A, Copper transporter, Glutathione reductase, Vacuolar protein sorting-associated protein, HbMyb1 transcription factor, Conserved hypothetical protein and Hevea brasiliensis clone Y17H05 mRNA, respectively. F and R represent forward and reverse primers, respectively.

## Discussions

We reported here the identification and characterization of the genes associated with TPD in rubber tree. In our research, the subtraction efficiency was validated by the expression analyses of 18s rRNA gene (Figure 2). The clones displaying the same expression levels between TPD and healthy trees were effectively excluded by reverse Northern blot analysis (Figure 3). In addition, the validation of SSH data was further verified by the expression patterns of 20 genes selected for RT-PCR and real-time PCR analysis (Figure 5). These results indicated that the genes identified in our study differentially express between healthy and TPD trees, suggesting that they might be associated with TPD in rubber tree. To enrich the information of genes related to TPD, all 237 unique genes were deposited to the NCBI database. With the SSH method, Venkatachalam et al. identified 134 unique genes associated with TPD from *Hevea *latex [21]. Among these genes, only 21 were identified in our research. The few common genes may be a result of several factors, such as the use of different *Hevea *clones, tapping systems and stages of TPD, etc. Therefore, it was necessary to identify more genes related to TPD with different experimental materials. The molecular mechanism underlying TPD could be precisely elucidated only if enough the genes related to TPD were identified. Compared with the functional classification of TPD-related genes from Venkatachalam et al. [21], one obvious similarity between the two studies was that a large numbers of stress/defense response genes were upregulated in TPD tree (Figure 4). The upregulation of these genes might destroy the normal cellular metabolism and result in the occurrence of TPD in rubber tree.

### Potential ROS producing and scavenging pathways

ROS, as toxic molecules, are capable of injuring cells. 17 genes probably involved in producing and scavenging ROS were identified in this research (Additional file 1, 2 and Table 2). Two genes, SSH235 matching *OsRac1 *and SSH37 matching phospholipase C, were all upregulated in TPD tree. It was reported that the upregulation of *OsRac1 *and phospholipase C might induce ROS production in plants [24-26]. Intriguingly, a putative gene encoding cinnamoyl-CoA reductase (CCR), an effector of *OsRac1*, was also increased in TPD tree. The CCR activity and ROS production were enhanced in the transgenic cell cultures constitutively expressing active *OsRac1 *[27]. Therefore, the three genes mentioned above might play the similar functions in rubber tree. On the other hand, the putative ROS-scavenging genes, such as thioredoxin fold, thioredoxin H-type, oxidoreductase, glutaredoxin (GLR), catalase (CAT), SOD, metal ion binding protein, ascorbate peroxidase (APX), glutathione reductase (GR) and cytochrome C oxidase, were all downregulated in TPD tree. Among of these genes, the decreasing expression of metal ion binding protein and thioredoxin H-type was also reported in TPD tree by Venkatachalam et al. [21]. In addition, the expression patterns of GR in TPD and healthy trees were further identified by RT-PCR and real-time PCR analyses (Figure 5). The expression profiles of the genes in ROS network probably break the balance between producing and scavenging ROS, which results in the accumulation and burst of ROS [28]. Faridah et al. reported that an uncompensated oxidative stress might be involved in the onset of TPD [12]. In TPD tree, the NAD(P)H oxidase activities increased [11], whereas the levels of variable peroxidase and SOD decreased [16]. Based on the above analyses, the accumulation and burst of ROS might occur in TPD tree. Being consistent with the hypothesis, the genes, encoding late embryogenesis abundant protein, S-adenosylmethionine-dependent methyltransferase and alcohol dehydrogenase, were all upregulated in TPD tree. The upregulation of three genes played important roles in protecting cells against oxidative stress [29-33]. A gene with high similarity to TCTP was downregulated in TPD tree, which was in agreement with the result from Venkatachalam et al. [21]. The transgenic *Escherichia coli *overexpressing *rBmTCTP *in vivo was subjected to oxidative stress [34]. Moreover, the genes encoding LEA 3 and chitinase were upregulated in TPD tree [21]. It was reported that LEA 3 and chitinase were involved in protecting macromolecules and membranes against oxidative stress [29].

**Table 2:** The genes associated with TPD involved in putative pathways

Putative pathways	**Putative genes**^**a**^
ROS	*Rac1*, Cinnamoyl-CoA reductase, Thioredoxin fold, Thioredoxin H-type, GLR, Phospholipase C, Oxidoreductase, Alcohol dehydrogenase, CAT, MnSOD, APX, Cytochrome C oxidase, Late embryogenesis abundant protein, GR, S-adenosylmethionine-dependent methyltransferase, TCTP, Metal ion binding protein.
UPP	Putative ubiquitin ligase, cullin, Ubiquitin-conjugating enzyme, Ubiquitin, Ubiquitin carrier protein, 26S protease regulatory subunit 6b, Ubiquitin-like protein, Ubiquitin-conjugating enzyme rad6, Ubiquitin-protein ligase.
PCD	Metacaspase type II, CED-12, Farnesyltransferase alpha subunit, Translation initiation factor 5A, Aquaporin, Translation elongation factor 1-alpha, Senescence-associated proteins (3), DNA topoisomerase II, ADP-ribosylation factor, Heat shock proteins (2), Cysteine desulfurylase, Phosphatase 2c, Speckle-type POZ protein, *Mlo*, *HbMyb1*.
RB	5-phosphomevelonate kinase, Rubber elongation factor (2), Small rubber particle, *HbTOM20*.

^a ^The numbers in the brackets represent the gene numbers identified in the pathway.

### Potential ubiquitin proteasome pathways

The cell response to stress is complex and is often concomitant with damage to a number of biomolecules including proteins [35-37]; therefore, it is conceivable that some repair mechanisms such as ubiquitin proteasome pathway (UPP) are involved in the cell response to oxidative stress. For example, the exposure to oxidative stress could produce high levels of damaged proteins that could be, at least in part, eliminated by the UPP [38,39]. It was reported that the oxidative stress could induce the expression of the major components involved in UPP [40-42]. In this research, nine putative genes involved in the UPP were identified in TPD tree (Additional file 1 and Table 2); the expression of putative genes encoding E2 (ubiquitin-carrier or conjugating proteins), E3 (ubiquitin-protein ligase), 26S protease regulatory subunit 6b, ubiquitin, ubiquitin-like gene and cullin forming E3 ubiquitin ligase complexes were all upregulated in TPD tree. Moreover, the expression patterns of E3 and cullin were further validated by RT-PCR and real-time PCR analyses (Figure 5). UPP is the major system for protein degradation [43,44], so the upregulation of key genes involved in UPP might facilitate the protein degradation in TPD tree. Interestingly, Venkatachalam et al. also identified the expression alteration of genes related to protein degradation in TPD tree [21]. Being consistent with our speculation, the low protein content in TPD tree was reported by Fan and Yang [10]. It is well-known that proteins play a vital role in maintaining normal cellular processes, whereas the low protein content caused by protein degradation in TPD tree might affect some of normal processes, such as PCD, rubber biosynthesis, cell biogenesis, etc. Oxidative stress is a prerequisite for inducing UPP; and therefore the upregulation of the key genes associated with UPP further suggested the accumulation and burst of ROS in TPD tree.

### Potential PCD pathways

Besides the induction of UPP, ROS can also initiate the programmed cell death process [45-47]. It is crucial for induction of PCD to produce more ROS and decrease ROS scavenging capacities [48,49]. In TPD tree, the expression patterns of genes involved in scavenging and producing ROS corresponded with the condition of initiating PCD (Additional file 1 and 2). Interestingly, twelve genes likely involved in inducing or executing PCD were identified in this research (Additional file 1, 2 and Table 2). A gene encoding putative metacaspase type II was upregulated in TPD tree, and its expression was verified by RT-PCR and real-time PCR analyses (Figure 5). In *Arabidopsis*, the upregulation of metacaspase type II could result in activating the downstream proteases, whereas proteases are required to mediate cell death via oxidative stress [50]. The gene similar to *CED-12 *was increased in TPD tree. The engulfment of cells undergoing apoptosis is the ultimate objective of the apoptotic program, and *CED-12 *is required for engulfment of dying cells and cell migrations [51-54]. *HbMyb1 *was decreased in TPD tree, and its expression profiles were verified by RT-PCR and real-time PCR (Figure 5). The downregulation of *HbMyb1 *might induce PCD in rubber tree [20,21]. The putative gene with homology to farnesyltransferase alpha subunit (FTase-alpha) was decreased in TPD tree; the antisense FTase-alpha resulted in cell death in Rat-2/H-ras cells [55]. A gene matching translation initiation factor 5A (*eIF5A*) was reduced in TPD tree; the previous findings indicated that *eIF5A *negatively regulated programmed cell death [56-61]. An aquaporin-like gene was downregulated in TPD tree; the transgenic lines downregulating aquaporin showed small plants, early senescence and lesion formation in *Arabidoposis *[62]. A gene with high similar to elongation factor 1-alpha was identified and it was upregulated in TPD tree; the gene plays an important role in executing the apoptotic program under oxidative stress [63,64]. Three genes encoding putative senescence-associated protein were all induced in TPD tree; the upregulation of senescence-associated protein (GO349117) was verified by RT-PCR and real-time PCR analysis (Figure 5), which was in agreement with the result of Venkatachalam et al. [21]. It was reported that the increased expression of senescence-associated proteins predispose tissue to senescence and cell death [65,66]. A gene being high similarity with a DNA topoisomerase II was upregulated in TPD tree, and this gene had the potential to trigger cell death pathways [67-69]. The ADP-ribosylation factor-like gene was increased in TPD tree. The transgenic tobacco overexpressing ADP-ribosylation factor showed cell death [70]. Due to high identifies to the known genes, the putative genes described above might induce or execute PCD in TPD tree.

PCD has been defined as a sequence of events controlling and organizing the cell destruction [71]. In this research, six genes probably protecting or inhibiting cells from PCD destruction were identified in TPD tree (Additional file 1, 2 and Table 2). Two homologies with heat shock protein were upregulated in TPD tree. Interestingly, Venkatachalam et al. also reported that two members of the heat shock proteins were increased in TPD tree [21]. It was found that heat shock protein protected cell form apoptosis [72]. A gene similar to cysteine desulfurylase was enhanced in TPD tree; The plants with reduced cysteine desulfurylase expression exhibited a disorganized chloroplast structure, stunted growth and eventually became necrotic and died before seed set [73]. The expression of a phosphatase 2c-like gene was induced in TPD tree. In plants and human, the phosphatase 2c negatively regulated cell death and oncogenicity, respectively [74,75]. A gene matching speckle-type POZ protein (SPOP) was downregulated in TPD tree, and HeLa cells overexpressing *SPOP *underwent apoptosis [76]. The expression of a gene with homology to the barley *Mlo *gene was elevated in TPD tree. In barley, *Mlo *transcripts were increased during leaf senescence, suggesting that *Mlo *might play important roles in preventing cell death [77]. Chen et al. and Venkatachalam et al. all suggested that PCD might occur at the onset of TPD [20-22]. In addition, the typical characters of PCD, such as DNA laddering, chromatin condensation, nuclear membrane blebbing and cytoplasm shrinkage, etc, were detected in TPD tree (communicated with Prof. Shiqing Peng). PCD is accepted as a fundamental cellular process in plants. It is involved in defense, development and response to stress. During the PCD process, the rubber tree might try to keep itself survival by executing the partial cell death. In fact, the phenotypes of cell death always appear on the tapping panel of TPD tree but not healthy one.

### Potential rubber biosynthesis pathways

Natural rubber is synthesized via the mevalonate (MVA) pathway in *Hevea brasiliensis *[78-80]. Besides the genes involved in the MVA pathway, rubber elongation factor, *HbTOM20 *and small rubber particle protein also play important roles in rubber biosynthesis [22,81-84]. In this study, two rubber elongation factors, *HbTOM20*, one small rubber particle protein and one 5-phosphomevelonate kinase were all downregulated in TPD tree (Additional file 2 and Table 2); the expression profiles of small rubber particle protein between TPD and healthy tree were further identified by RT-PCR and real-time PCR analysis (Figure 5). Besides the above genes, Venkatachalam et al. reported that a rubber biosynthetic gene, *HbHMD-CoA*, was upregulated in TPD tree [21]. Although the exact roles of those genes in the occurrence of TPD are not clear, they may be associated with the decreased latex biosynthesis and/or flow in TPD tree.

## Conclusions

Altogether, the genes associated with TPD were identified and their characterizations were further analyzed in the paper. Among 237 unique genes, 205 were firstly reported to be related to TPD in rubber tree; these genes laid the foundations for unraveling the molecular mechanisms involved in TPD. This result also demonstrates that it is necessary to identify the genes associated with TPD from different clones, tapping systems and stages of TPD. Of different functional categories, the large numbers of genes related to TPD were associated with transcription and post-transcription, metabolism and energy, protein metabolism or stress/defense response. In addition, the characterization and expression of the genes related to TPD suggested that ROS producing and scavenging, UPP, PCD and RB might play important roles in TPD occurrence, which provides new insights into understanding TPD in rubber tree.

## Methods

### Plant material

The RY8-79, a high-yielding clone but prone to TPD, was planted at the experimental farm of Chinese Academy of Tropical Agricultural Sciences. In this experiment, the latex has been harvested for the past 11 years. During the past 11 years, the rubber trees were regularly tapped with a standard tapping system (S/2 d/4, i.e. half spiral cut tapped at the fourth daily frequency). Besides, 1.0% ethephon was applied to stimulate latex yield two days before tapping, and the stimulation frequency was once three tappings. The trees affected by TPD syndrome were still tapped along with healthy trees to maintain uniform conditions until sample collection. The fresh latex samples were separately collected and pooled from five healthy and TPD trees, and immediately frozen in liquid nitrogen for total RNA extraction.

### RNA isolation

Total RNA was extracted from the latex samples of healthy and TPD trees according to the method of Tang et al. [85]. The RNA quantity and quality were determined by the spectrophotometrically.

### Construction of SSH cDNA libraries

For PCR-select cDNA subtraction, Poly(A)^+ ^mRNA was purified from total RNA with an Oligotex™-dT30 mRNA Purification Kit (Takara). SSH was performed using the PCR-Select™ cDNA subtraction kit (Clontech, CA, USA) according to the manufacturer's protocol and the method of Diatchenko et al. [86]. For forward library, the cDNA from healthy and TPD trees was used as "tester" and "driver", respectively. On the contrary, the cDNA from TPD and healthy trees was separately used as "tester" and "driver" in reverse one. The double-stranded cDNAs were separately synthesized from 2 ug Poly(A)^+ ^RNA samples generated from healthy and TPD trees. The driver and tester cDNAs from forward and reverse libraries were separately digested with *Rsa*I and then purified. The digested tester cDNA was subdivided into two portions, and each was separately ligated to different adaptors (adaptor 1 or adaptor 2R) provided by the manufacturer. After the ligation, the resulting cDNAs (tester cDNAs ligated with adaptors) were divided into two populations: one for subtraction study and the other for the evaluation of subtraction efficiency.

Two hybridizations were then performed. In the first hybridization, an excess of driver was added to each tester samples, leading to the enrichment of differently expressed sequences. During the second hybridization, the two primary hybridization samples were mixed together to form new double-stranded hybrids with different ends. Fresh denatured driver cDNA was added to further enrich differentially expressed sequences. After two hybridizations, the resulting annealed material was used as the PCR template. The primary PCR was performed with the following parameters: 94°C for 25 s followed by 27 cycles of 94°C for 30 s, 68°C for 30 s and 72°C for 1.5 min, and then 72°C extension for 7 min. The primary PCR products were diluted 10-fold and used as the template in secondary PCR. The secondary PCR was performed for 14 cycles with the same parameters as the primary one. The subtraction efficiency was evaluated by PCR reaction with the primers of rubber 18s rRNA. The PCR products from subtracted samples were inserted into the pMD18-T vector (Takara) and then transferred into chemically competent *E. coli *(DH5a) cells to generate SSH libraries. The transformants were planted on LB agar plates with 100 ug/ml ampicillin, 40 ug/ml 5-Bromo-4-chloro-3-indolyl β-D-galactopyranoside (X-Gal) and 1 mM isopropyl-beta-D-thiogalactopyranoside (IPTG) for blue-white screening. The white colonies were picked and cultured at 37°C in LB liquid culture medium with 100 ug/ml ampicillin. The glycerol stocks of bacterial cultures were frozen in liquid nitrogen and stored at -80°C.

### Amplification of cDNA insertions

All recombinant clones from the two subtracted libraries were picked and cultured overnight in LB liquid culture medium with 100 ug/ml ampicillin. The cDNA inserts were amplified with nested PCR primers (F 5'-tcgagcggccgcccgggcaggt-3' and R 5'-agcgtggtcgcggccgaggt-3'). The PCR amplifying conditions were as follows: 94°C for 5 min; 30 cycles of (94°C for 30 s, 68°C for 30 s and 72°C for 2 min); and a final extension at 72°C for 10 min. The PCR products were then electrophoresed on 1.2% agarose gel.

### Differential screening of the cDNA clones by reverse Northern analysis

To identify and select the differentially expressed clones, reverse Northern bolt was performed with labeled cDNA probes. The subtracted and unsubtracted cDNAs were labeled with *a*-^32^P-dCTP using Random Primer DNA Labeling Kit (Takara). The labeled probes were obtained and purified on Sephadex G50 columns. The denatured PCR products of inserts (about 10 ng) were spotted onto nylon membranes. The membranes were incubated in standard prehybridization solution at 65°C for 3 h and then hybridized with *a*-^32^P-labeled probes at 65°C for 12 h. Following hybridization and sequential washing, the radioactive membranes were exposed to x-ray film. The clones, only hybridizing with the labeled tester probes or indicating at least threefold signal with the labeled tester probes than the labeled driver ones, were selected to sequence.

### DNA sequencing and sequence analysis

The differentially expressed clones in two cDNA libraries were selected and sequenced. Raw sequence trace files were performed by DNA Sequencing Analysis Software 5.1 (Applied Biosystems) to obtain base-calling with quality scores. Low quality (quality score < 16), short (< 100 bp), vector and adaptor sequences were removed by Lucy program [87]. All unique ESTs were searched NCBI database with basic local alignment search tool (BLAST) program [88]. The functional categories of all unique ESTs were performed according to GO [23].

### RT-PCR and Real-Time PCR analysis

The cDNA synthesis was performed using 2 ug total RNA according to the manufacture's protocol (Invitrogen, USA). All RT-PCR experiments described here were repeated at least three times using independent cDNA samples. In each PCR reaction, the gene-specific primers were used, and rubber 18s rRNA gene was used as the internal reference. All primers used for expression analysis are provided in Table 1. RT-PCR was performed with the following parameters: 94°C for 4 min followed by 30 cycles of 94°C for 30 s, 58°C for 30 s and 72°C for 1 min. The final extension was performed at 72°C for 10 min. The RT-PCR products were analyzed by electrophoresis in 1.2% agarose gels.

For real-time PCR analysis, the RNA samples were prepared from three biological repetitions; one was used to construct SSH library; two were different from those used for SSH. The real-time PCR reactions were performed with ABI 7900 real-time PCR system. The SYBR Premix Ex Taq kit (Takara) was used according to the manufacturer's protocol. The expression level of each gene was normalized against the 18S rRNA gene. The cDNA samples were prepared with a series of 100-fold dilutions, and the amplification efficiency of each gene was adjusted to be equal to 18S rRNA. For each target gene, the PCR reactions were carried out in triplicate. The relative expression values were calculated from three biological replicates using a modified 2^-ΔΔC^T method [89].

## Abbreviations

APX: ascorbate peroxidase; BLAST: basic local alignment search tool; CAT: catalase; CCR: cinnamoyl-CoA reductase; DDRT-PCR: differential display reverse transcription PCR; EST: expressed sequence tag; GO: Gene Ontology; GLR: glutaredoxin; IPTG: isopropyl-beta-D-thiogalactopyranoside; MVA: mevalonate; GR: glutathione reductase; NAD(P)H: oxidized and reduced nicotinamide-adenine dinucleotide phosphate; RB: rubber biosynthesis; ROS: reactive oxygen species; RT-PCR: reverse-transcription PCR; SPOP: speckle-type POZ protein; SOD: superoxide dismutase; SSH: suppression subtractive hybridization; TPD: tapping panel dryness; TCTP: translationally controlled tumor protein; UPP: ubiquitin proteasome pathway; X-Gal: 5-Bromo-4-chloro-3-indolyl β-D-galactopyranoside.

## Authors' contributions

All the authors read and approved the final manuscript. DL supervised the experiments, and carried out RNA extraction, library construction and data analyses. In addition, he drafted and revised the manuscript. ZD prepared clones for sequencing, performed blast analyses and functional classification of ESTs, RT-PCR and real-time PCR analyses. CC collected plant materials, and performed RNA extraction, clone selection and PCR identification. ZX carried out reverse Northern analysis, PCR detection and data analyses. MW and PH collected plant materials, performed RNA extraction and library construction. SC supervised the research.

## Supplementary Material

Additional file 1 **The identification of non-redundant clones upregulated in TPD trees from the reverse SSH library**. The file contains functional classification, SSH clone ID, cDNA insert size, GeneBank accession no., putative identify and E-value of non-redundant clones from the reverse SSH library. Click here for file

Additional file 2 **The identification of non-redundant clones downregulated in TPD trees from the forward SSH library**. The file contains functional classification, SSH clone ID, cDNA insert size, GeneBank accession no., putative identify and E-value of non-redundant clones from the forward SSH library. Click here for file
